# Supplementary material for: The ubiquitin-like modifier FAT10 is degraded by the 20S proteasome in vitro but not in cellulo
Source: Life Sci Alliance. 2023 Apr 3;6(6):e202201760. doi: 10.26508/lsa.202201760 (PMC10070814; doi:10.26508/lsa.202201760)
Supplement: Supplementary file 1 [file LSA-2022-01760_TableS1.docx]

| **Target** | **Host** | **Isotype** | **Clone** | **Supplier** | **Identifier, Catalog-ID** |
| --- | --- | --- | --- | --- | --- |
| **Flag** | Mouse | IgG1 | M2 | Sigma Aldrich | F1804 |
| **FAT10** | Mouse | IgG2a | 4F1 | Aichem et al., 2010 (70) | BML-PW0765; RRID:AB_11000253 |
| **GAPDH** | Rabbit | IgG | Polyclonal | Sigma Aldrich | G9545 |
| **MCP231 (α1-7)** | Mouse | IgG1 | Monoclonal | Abcam | ab22674 |
| **PSMC1 (Rpt2)** | Rabbit | IgG | Polyclonal | Bethyl | A303-821A-M |
| **Rpt1** | Mouse |  | 4C10-2C8 | Biozol | USB-131963-HRP |
| **Rpt6** | Rabbit |  | polyclonal | Enzo Life Sciences | BML-PW8320 |
| **TBP1 (Rpt5)** | Rabbit | IgG | Polyclonal | Bethyl | A303-538A-T |
| **Ubiquitin** | Mouse | IgG1 | FK2 Monoclonal | Enzo Life Sciences | BML-PW8810 |

**Table S1. List of antibodies.** Details of primary antibodies used for western blotting.
